# Multiscale variability in nutrients and secondary metabolites in a bat‐dispersed neotropical fruit

**DOI:** 10.1002/ece3.10453

**Published:** 2023-09-02

**Authors:** Mariana Gelambi, Susan R. Whitehead

**Affiliations:** ^1^ Department of Biological Sciences Virginia Polytechnic Institute and State University Blacksburg Virginia USA; ^2^ La Selva Biological Station Organization for Tropical Studies Puerto Viejo de Sarapiquí Heredia Province Costa Rica

**Keywords:** chemical ecology, Costa Rica, nutrient–toxin titration hypothesis, nutritional ecology, *Piper sancti‐felicis*, removal‐rate hypothesis, specialized metabolites

## Abstract

Ripe fleshy fruits contain not only nutrients but also a diverse array of secondary metabolites. Nutrients serve as a reward for mutualists, whereas defensive metabolites protect the fruit against pests and predators. The composition of these chemical traits is highly variable, both across different plants and even within repeating structures on the same individual plant. This intraspecific and intraindividual variation has important fitness consequences for both plants and animals, yet patterns of variation and covariation in nutrients and secondary metabolites are not well understood, especially at smaller scales. Here, we investigate the multiscale variation and covariation between nutrients and defensive metabolites in *Piper sancti‐felicis* ripe fruits. Means and measures of variation of sugars, proteins, phenolics, and alkenylphenols vary greatly among plants, and at least 50% of the trait variation occurs at the intraindividual level. Also, we found that proteins, but not sugars, were correlated with phenolics and alkenylphenols at multiple scales, suggesting trait variation in protein content may be more constrained than sugars. Our findings emphasize the importance of examining patterns across scales and provide the groundwork to better understand how complex patterns of variation and covariation in nutrients and defensive metabolites shape ecological interactions surrounding fruits.

## Graphical abstract

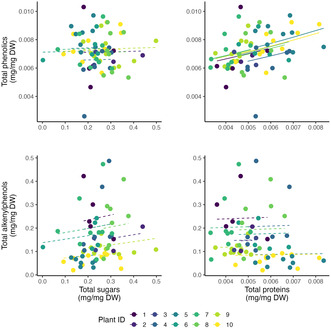

## 1 INTRODUCTION

During foraging, plant consumers encounter enormous biochemical variation, both in nutrients and in secondary metabolites, a structurally heterogeneous group of molecules ubiquitous in plants (Kessler & Kalske, 2018; Schoonhoven et al., 2005). Secondary metabolites have multiple roles in plant tissues, but they have been primarily studied in the context of defenses against antagonists. Defensive secondary metabolites negatively affect the performance of plant consumers by acting as toxins or reducing nutrient availability (Kessler & Kalske, 2018; Schoonhoven et al., 2005). Numerous studies have sought to understand how variation and covariation in these key plant chemical traits influence foraging (Behmer et al., 2002; Westerband et al., 2021). Although most have focused on the interspecific (among‐species) or intraspecific (within‐species) scale, recent evidence suggests that much of the trait variation in plants occurs at even smaller scales—across repeating structures on the same individual plant (Herrera, 2017; Sobral et al., 2019; Wetzel & Meek, 2019). This intraindividual (within‐plant) variation may have important fitness consequences for both plants and animals. Intraindividual variation per se can be a trait under selection driven by abiotic and biotic factors (Herrera, 2009; Shimada et al., 2015; Sobral et al., 2014). However, patterns of variation and covariation in nutrients and secondary metabolites at this scale are not well described, especially for plant organs other than leaves.

Ripe fleshy fruits often contain high nutrient concentration as a reward for mutualistic seed dispersers (Corlett, 2011; Jordano, 2000) and complex mixtures of defensive metabolites that protect the pulp and seeds against potential pests and pathogens (Cipollini, 2000; Cipollini & Levey, 1997a). In some fleshy fruits, the occurrence of defensive metabolites results in an ecological cost, where defensive metabolites deter not only antagonists but also legitimate seed dispersers (Cipollini, 2000). The strength of these trade‐offs depends on a variety of factors, including the patterns of association between defensive metabolites and nutritional rewards (i.e., sugars, proteins, and lipids) in fruit pulp. Three main adaptive hypotheses have been proposed to explain the association between the concentration of defensive metabolites and nutrients in fruit pulp and how the association mediates biotic interactions (Cipollini, 2000; Cipollini & Levey, 1997a; Cipollini & Stiles, 1992). The “removal‐rate hypothesis” predicts a negative association between defenses and nutrients, arguing that frugivores will quickly remove high‐nutrient fruits and, thus, these fruits do not require high concentrations of defensive compounds. In contrast, the “nutrient–toxin titration” and “relative risk” hypotheses predict a positive association, though their mechanisms are distinct (Cipollini, 2000). The “nutrient‐toxin titration” model argues that mutualistic and antagonistic frugivores will consume high‐nutrient fruits, as nutrients can compensate for any negative effects caused by high concentrations of defensive metabolites (Cipollini, 2000). The “relative risk” model suggests that high nutrients fruits should contain higher levels of defensive metabolites because they are at higher risk of attack from microbial and invertebrate pests.

The above hypotheses predict positive or negative patterns of associations between nutrients and secondary metabolites at the interspecific scale, but they have also been applied at the intraspecific scale (Cipollini & Levey, 1997b; Izhaki et al., 2002). The limited available evidence suggests that these positive or negative associations may be scale‐dependent. At the interspecific scale, evidence from fruit glycoalkaloids (Cipollini & Levey, 1997b) and phenolic compounds (Cazetta et al., 2008; Schaefer et al., 2003) support the “removal‐rate hypothesis.” Conversely, at the intraspecific scale, evidence from emodin (an anthraquinone) supports the “nutrient‐toxin titration hypothesis” (Izhaki et al., 2002). To our knowledge, the association between nutrients and defensive metabolites has not been tested at the intraindividual (among fruits within plants) scale, even though intraindividual variation in fruit traits has important consequences for plant interactions (Herrera, 2017; Palacio et al., 2017; Sobral et al., 2013; Wetzel et al., 2016; Wetzel & Meek, 2019) and trait correlations are often scale‐dependent (Agrawal, 2020). As most studies in chemical ecology have focused on reporting a single class of chemical traits at a single scale, we have a limited understanding of patterns of covariation between nutrients and secondary metabolites and the extent to which they are scale‐dependent.

In our study, we explored the multiscale variability of nutrients and defensive metabolites in ripe infructescences of a bat‐dispersed tropical shrub, *Piper sancti‐felicis* Trel. (Piperaceae). Specifically, our objectives were to: (1) Describe the amount and pattern of intraspecific (also referred to in the literature as ‘interindividual’) and intraindividual variation in two key groups of nutrients, sugars and proteins, and two classes of defensive metabolites, phenolics and alkenylphenols; and (2) Determine whether covariation between nutrients and defensive metabolites at the intraspecific and intraindividual scales support the “removal‐rate” or “nutrient‐toxin titration/relative risk hypotheses.” We chose to focus on phenolics and alkenylphenols because they are the major groups of secondary metabolites found in *P. sancti‐felicis* and are known to serve a variety of adaptive roles. Phenolics are ubiquitous in plants and have been widely considered defensive metabolites against herbivores (Kumar et al., 2020) and deterrents to frugivores (Cazetta et al., 2008; Zungu & Downs, 2015), but may have multiple other functions, such as protection against abiotic stress (Naikoo et al., 2019). Alkenylphenols are less common metabolites and appear to have more specialized roles in fruits. Alkenylphenols have been reported in several *Piper* spp. (Yoshida et al., 2018) and occur in high concentrations in the fruit pulp (but not leaves or seeds) of *P. sancti‐felicis* (Maynard et al., 2020). Alkenylphenols from ripe *P. sancti‐felicis* fruits strongly reduce the growth rates of potential fungal pathogens (Maynard et al., 2020). Alkenylphenols also deter bats, the main dispersers of *Piper* fruits, but have no detectable effects on ants, which commonly act as secondary dispersers (Clemente & Whitehead, 2020; Maynard et al., 2020). Thus, both phenolics and alkenylphenols likely have important defensive roles in fruits and broad ecological significance for seed dispersal. Understanding the association between nutrients and fruit‐defensive metabolites is crucial to describe the array of resources that plant consumers encounter in nature, and ultimately improve our understanding of the mechanisms that shape foraging patterns.

## 2 METHODS

### 2.1 Study site and species

Fruit collection for our study was conducted at La Selva Biological Station (LSBS), province of Heredia, Costa Rica, between June and July 2021. LSBS consists of 1536 hectares of lowland protected area (McDade et al., 1994) managed by the Organization for Tropical Studies (OTS). LSBS is a mosaic of primary and secondary forests connected to Braulio Carrillo National Park (McDade et al., 1994). LSBS supports high levels of plant and animal diversity, including more than 60 *Piper* spp. (OTS, 2020). *Piper*, a diverse genus of flowering plants in the pepper family (Piperaceae), is characterized by spike‐like infructescences with small, single‐seeded fruits organized around a central rachis. Bats from the genus *Carollia* are the main seed dispersers of *Piper* spp.

Our focal species, *P. sancti‐felicis*, is an especially abundant *Piper* species in Central American forests, and one of the most important food items of *Carollia* spp. in the study site (Maynard et al., 2020; Santana et al., 2021). *Piper* fruits are a unique system to test the “removal‐rate hypothesis” versus the “nutrient‐toxin titration/relative risk hypotheses” because of the notably high removal rates of ripe *Piper* spp. fruits (Maynard et al., 2020; Thies & Kalko, 2004 ). The ripening process occurs rapidly throughout a single afternoon. All fruits on an infructescence ripen together, and, once ripe, entire infructescences (hereafter “fruits”) are quickly removed from the plant by bats, most often on the same night of ripening (Maynard et al., 2020; Thies & Kalko, 2004).

### 2.2 Fruit collection and processing

We collected the fruits under a Costa Rican permit issued by CONAGEBIO (Comisión Nacional para la Gestión de la Biodiversidad, resolution number R‐007‐2021‐OT‐CONAGEBIO). We collected 67 ripe fruits from 10 *P. sancti‐felicis* shrubs (2–11 fruits per shrub) in sites close to the lab clearing and close to the entrance of LSBS. As ripe *Piper* spp. fruits are avidly consumed by frugivores, we covered unripe fruits with mesh bags and checked the covered fruits daily early in the morning. We identified ripe fruits by their soft texture, ease of detachment from the branch, and darker coloration. When ripe, we collected the covered fruits and stored them at −80°C until we were able to perform chemical analysis, except for brief transport from Costa Rica to Virginia, USA on dry ice.

To obtain dried fruit pulp, we lyophilized frozen fruits for 72 h in a freeze dryer (SP Industries, Inc.). Once the fruits were fully dried, we used a mortar and pestle to carefully pulverize the fruit pulp and not the seeds. Once we obtained a mix of a fine powder and seeds, we sieved the plant material to separate the pulp from the seeds. We discarded the fruit rachis because it is not consumed (Bizerril & Raw, 1998). We performed all the analyses described below for each individual fruit.

### 2.3 Sugar extraction and quantification

We modified the extraction described in Maness (2010). We weighed 10 mg of dried pulp and added 1 mL of 80% ethanol as the extraction solvent. Subsequently, we sonicated the samples for 20 min. In a water bath, we heated the samples for 15 min at 85°C and centrifuged the homogenates at 10,000× *g* for 10 min at room temperature. We performed the extraction twice. The supernatants from the two extractions were combined and we quantified the total concentration of d‐glucose and d‐fructose using a commercial kit (Megazyme, K‐SUFRG) adapted to a 96‐well plate. Each sample was extracted and analyzed in duplicate, and the two duplicate measures were averaged for all subsequent analyses. Other significant sugars for frugivore foraging, such as sucrose, were not quantified due to the limited amount of dried pulp available per individual fruit.

### 2.4 Protein extraction and quantification

We modified the extraction in Bonjoch and Tamayo (2001) to extract and quantify protein from plant tissues. We weighed 10 mg of dried pulp and added 1 mL of an extraction solvent consisting of 0.05 M Tris buffer (pH 8.0), ascorbic acid 0.1% (w/v), cysteine hydrochloride 0.1% (w/v), polyethylene glycol (1%), citric acid monohydrate 0.1% (w/v), and 2‐mercaptoethanol 0.008% (v/v). We sonicated the samples for 20 min and centrifuged the homogenates at 10,000× *g* for 10 min at 4°C. We then quantified the total amount of protein in the supernatant using a commercial colorimetric assay (Bio‐Rad, 5000002) based on the Bradford method (Bonjoch & Tamayo, 2001) adapted to 96‐well plates. We used a standard curve of bovine serum albumin to calculate total protein concentration. Every sample was extracted and analyzed in duplicate as above.

### 2.5 Total phenolic extraction and quantification

We followed the extraction and quantification of total phenolics described by Ainsworth and Gillespie (2007) using the Folin–Ciocalteu reagent. We weighed 25 mg of dried pulp and added 1 mL of 80% methanol as the extraction solvent. Subsequently, we sonicated the samples for 20 min and centrifuged the homogenates for 5 min at 10,000× *g*. We performed the extraction twice. The supernatants from the two extractions were combined and the reaction was set up with 80 μL of distilled water, 20 μL of samples, 200 μL of Folin reagent, and 800 μL of sodium carbonate solution (74.2 mg/mL). We added 200 μL of each sample into a 96‐well plate and measured the absorbance at 765 nm. We used a standard curve of gallic acid (GA) to calculate total phenolic concentration. Every sample was extracted and analyzed in duplicate as above.

### 2.6 Alkenylphenol extraction and quantification

We modified the extraction and gas chromatography–mass spectrometry (GC–MS) quantification of alkenylphenols described in Maynard et al. (2020). We weighed 10 mg of dried pulp and added 1 mL of ethanol as extraction solvent along with 10 μL of 20 μg/μL 4‐butylresorcinol (Sigma‐Aldrich) as the internal standard. Subsequently, we sonicated the samples for 20 min and centrifuged the homogenates at 10,000× *g* for 2 min at room temperature. We performed the extraction twice. The supernatants from the two extractions were combined and the ethanol was evaporated using a SpeedVac. In each tube, we added 600 μL of water and 200 μL of chloroform, vortexed for 5 min, and centrifuged for 2 min at 10,000× *g*. We discarded the aqueous layer, transferred 100 μL of the chloroform layer to a micro insert, evaporated, and resuspended in 50 μL of dichloromethane. We injected the samples into an Agilent 7820 gas chromatograph coupled with a 5977 mass spectrometer equipped with an HP5‐MS column (Agilent Technologies). To process alkenylphenol chromatography data, we converted Agilent files (.D) to AIA format (.CDF) using ChemStation and processed chromatography data using the R package metaMS (Wehrens et al., 2014). We saved every compound spectrum as .msp files, and using the NIST MS search 2.0, we identified individual alkenylphenols based on retention time (RT) and the similarities in the mass spectra reported by Maynard et al. (2020). We estimated total alkenylphenol concentrations based on the area of the internal standard. RT, average abundance, and frequency of detection of individual alkenylphenols are described in Table A1.

### 2.7 Statistical analysis

We performed all statistical analyses in R v. 4.1.3 (R Core Team, 2021). Multivariate analyses were performed using the vegan package (Oksanen et al., 2020), and generalized linear mixed models (GLMMs) and generalized linear models (GLMs) were performed using the glmmTMB package (Magnusson et al., 2017).

To describe the amount and pattern of intraspecific and intraindividual variation in nutrients and defensive metabolites (Objective 1), we used measures of central tendency (mean), dispersion (standard deviation, coefficient of variation), and skewness, calculated for each individual shrub and across shrubs. To evaluate whether different shrubs show overall differences in multivariate chemical composition or within‐shrub variation in composition, we performed a nonmetric multidimensional scaling (NMDS) on standardized values (*z*‐scores) using Euclidean distances. To test for statistically significant differences among shrubs, we conducted a permutational multivariate analysis of variance (PERMANOVA), using the function “adonis2()” with 999 permutations, and to test for homogeneity of variances, we used the function “betadisper()”, followed by an ANOVA to compare within‐shrub variation among shrubs. Finally, to quantify the relative amount of variation at the intraspecific and intraindividual scales, we conducted variance component analyses for glucose, fructose, total proteins, total phenolics, and total alkenylphenols. Using the approach described in Crawley (2012), we fitted one GLMM per chemical trait, using each trait as the response variable. Each model included a nested random effect coded as (1|PlantID/FruitID) as the only predictor variable. For each trait, we expressed the intraspecific and intraindividual variance components as a percentage of the total variance. With variance components analysis, the experimental variance is necessarily incorporated into the smallest sampling scale and thus would be included as part of the intraindividual variance. However, we expect experimental variance to be low for our samples, as the methods used here are highly reproducible and have a low coefficient of variation between samples (Malta & Liu, 2014; Okutucu et al., 2007).

To determine whether covariation between nutrients and defensive metabolites at the intraspecific and intraindividual scales support the “removal‐rate” or “nutrient‐toxin titration/relative risk hypotheses” (Objective 2), we first conducted correlation analyses for glucose, fructose, total proteins, total phenolics, total alkenylphenols, and the 10 individual alkenylphenol quantified using the function “cor()” and “cor.mtest()” to test for statistically significant Spearman's rank correlation coefficient among the chemical traits. The correlation matrix showed a set of significant correlations, such as those between glucose and fructose and between most of the individual alkenylphenols (Figure A2). To avoid multicollinearity problems in the models, we added glucose and fructose together, which we refer to here as “sugars,” and focused on total alkenylphenols rather than individual compounds. Then, to examine association patterns at the intraindividual scale, we constructed two separate GLMMs with a beta distribution and the logit link function, which is an appropriate distribution when response variables are percentages, proportions, and ratios (Smithson & Verkuilen, 2006). Utilizing GLMMs enables the incorporation of random effects to account for the hierarchical structure of our sampling design and address the nonindependence of observations within the data. One model used total phenolics as the response variable, while the other model used alkenylphenols. In both models, sugars and total proteins were included as predictor variables and plant ID as a random effect. To examine association patterns at the intraspecific level, we calculated the average values of each chemical trait per shrub and performed identical GLMs as described above but without any random effects. For each GLMM, we applied a Bonferroni correction for multiple testing and reported adjusted *p*‐values. To validate the models, we performed two different sensitivity analyses. At the interindividual level, we conducted a k‐fold cross‐validation (*k* = 10) and obtained an overall root mean square error (RMSE) of 0.0014, indicating that the model's predictions are closely aligned with the true values. At the intraspecific level, we systematically removed each individual from the dataset, confirming that, in most cases, the exclusion of specific individuals did not influence the reported *p*‐value.

## 3 RESULTS

### 3.1 Multiscale patterns of variation for nutrients and defensive metabolites (Objective 1)

**FIGURE 1:**
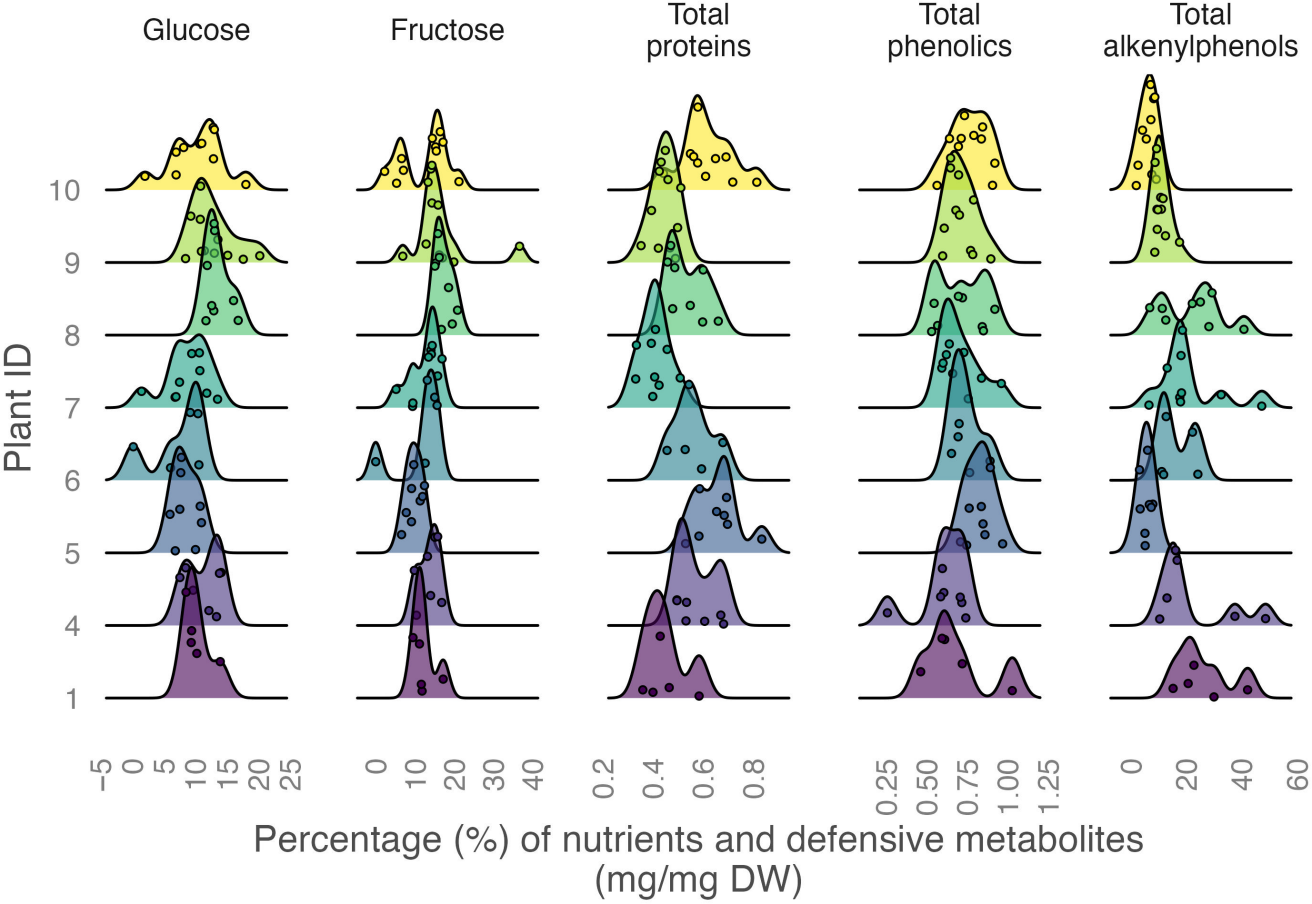
Ridgeline plot illustrating the distribution of the defensive metabolites (total and individual alkenylphenols, and total phenolics) and nutrients (glucose, fructose, and protein) in ripe *Piper sancti‐felicis* fruits collected from eight shrubs. The density curves display the distribution of each chemical trait within a shrub. Each point represents an individual fruit, colored by plant ID. Shrubs with a sample size of fewer than five fruits were excluded from the plot.

**FIGURE 2:**
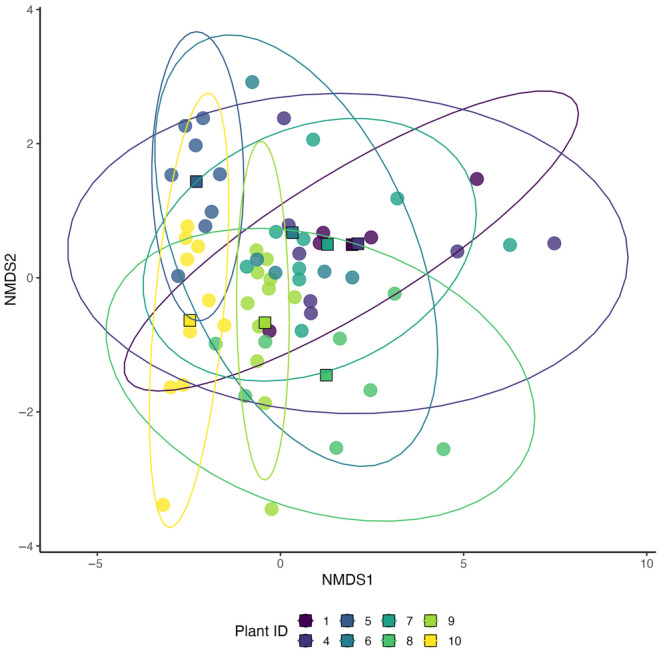
Nonmetric multidimensional scaling (NMDS) did not reveal distinct clusters (final stress = 0.139, PERMANOVA, *p* = .276) based on the composition of defensive metabolites (individual alkenylphenols and total phenolics) and nutrients (glucose, fructose, and protein). NMDS plot shows differences in the variance of chemical composition among shrubs (PERMDISP2, *p* = .006). Points represent one individual fruit, colored by plant ID. Squares represent group centroids, colored by plant ID. Shrubs with a sample size of fewer than five fruits were excluded from the plot.

**FIGURE 3:**
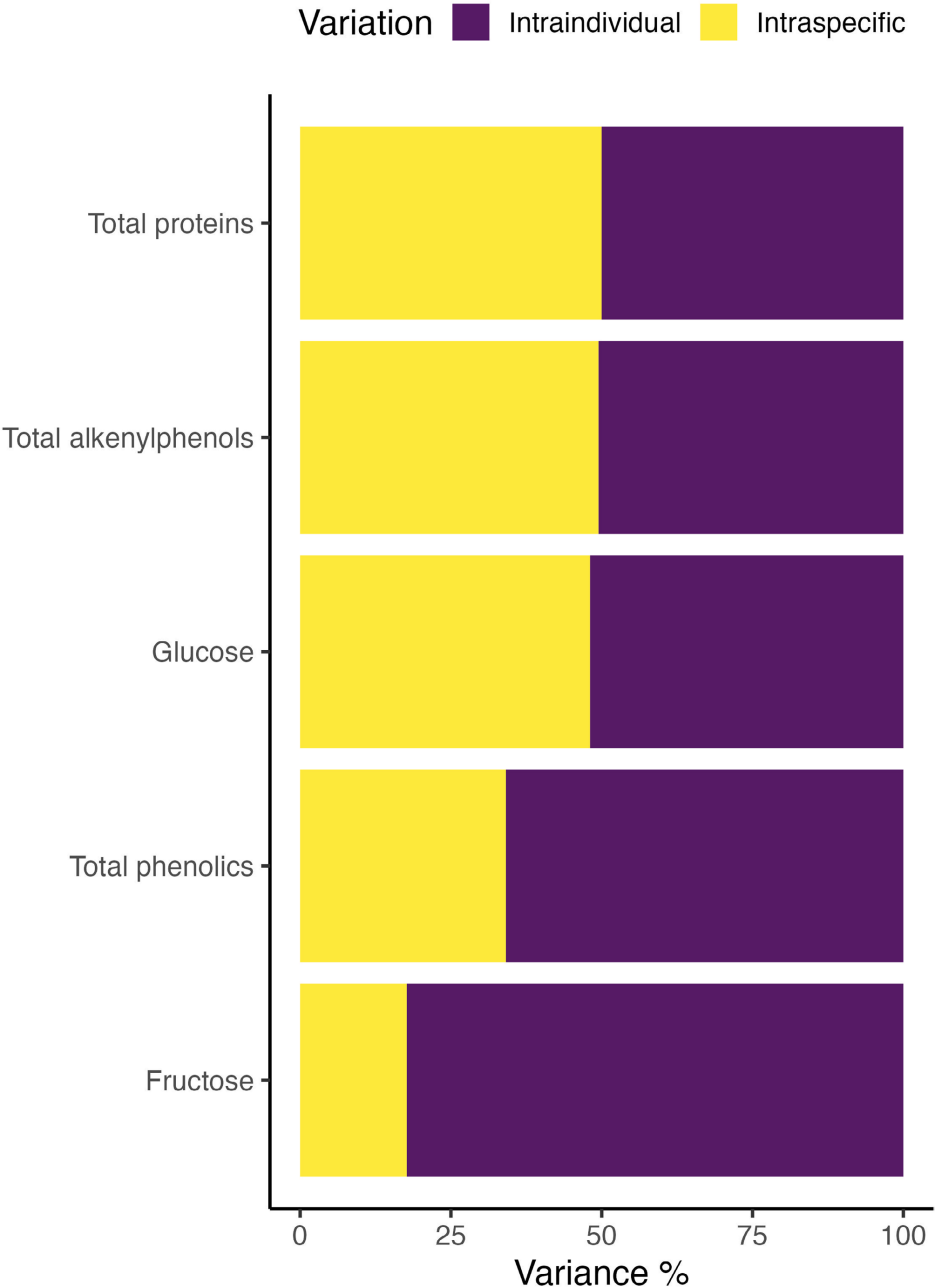
Graphical representation of variance components analysis for nutrients and defensive metabolites in ripe *Piper sancti‐felicis* fruits.

**FIGURE A1:**
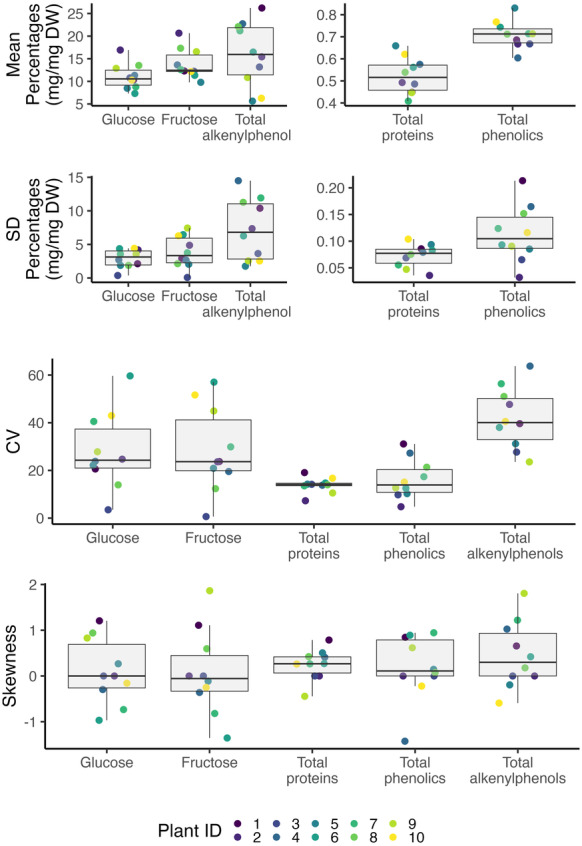
Summary statistics, including mean, standard deviation (SD), coefficient of variation (CV), and skewness of defensive metabolites (alkenylphenols and total phenolics) and nutrients (glucose, fructose, and protein) in multiple ripe *Piper sancti‐felicis* fruits collected from 10 shrubs.

**FIGURE A2:**
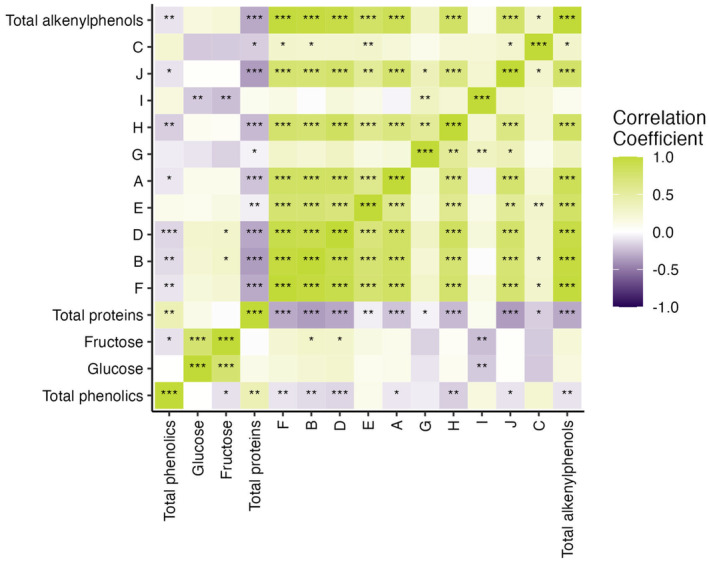
Correlation matrix plot of 15 chemical traits calculated using Spearman's rank correlation coefficient. Darker colors represent a stronger, either negative or positive, correlation. Letters represent individual alkenylphenols (see Table A1). Significance levels are denoted by asterisks, (***p < .001, **p < .01, *p < .05).

We aimed to describe the amount and pattern of intraspecific and intraindividual variation in two key groups of nutrients (sugars and proteins) and two classes of defensive metabolites (phenolics and alkenylphenols). First, we quantified glucose, fructose, total proteins, total phenolics, and total and individual alkenylphenols for 67 fruits from 10 shrubs (Figures 1, 2 and A1, Table 1). Among the quantified nutrients, fructose was found to be the most abundant in fruit pulp, followed by glucose, while proteins were the least abundant. Among the quantified defensive metabolites, alkenylphenols were the most abundant in fruit pulp, followed by total phenolics (Table 1). Among all fruits, fructose was the nutrient that showed the highest variation, followed by glucose and proteins. Alkenylphenols were the defensive metabolites that showed the highest variation, followed by total phenolics (Table 1). Individual shrubs do not show different average overall chemical composition (Figure 2, PERMANOVA, *p* = .276) but show differences in the amount of within‐shrub heterogeneity (Figure 2, PERMDISP2, *p* = .006). Differences across shrubs in the amount and pattern of within‐plant variation are also suggested for each chemical trait by the ridgeline plots, which show the full chemical trait distributions for each individual shrub (Figure 1). The variance components analysis showed that intraindividual variation in fruit chemical traits was similar to or even higher than intraspecific variation for all of the traits examined (Figure 3). For glucose, proteins, and alkenylphenols, there was a similar percentage of variance explained at the intraspecific and intraindividual scales, but for fructose and total phenolics, a higher percentage of variance was explained by the intraindividual level.

**TABLE 1:** Descriptive statistics of different chemical traits in individual ripe fruits of *Piper sancti‐felicis.*

Plant ID	Number of fruits analyzed	% Glucose, mg/mg DW	% Fructose, mg/mg DW	% Total proteins, mg/mg DW	% Total phenolics, mg/mg DW	% Total alkenylphenols, mg/mg DW
1	5	10.32 ± 2.13	12.30 ± 2.92	0.45 ± 0.09	0.69 ± 0.21	26.22 ± 10.38
2	2	16.92 ± 4.18	20.65 ± 4.88	0.49 ± 0.04	0.67 ± 0.03	15.42 ± 7.35
3	2	10.77 ± 0.37	12.24 ± 0.08	0.49 ± 0.07	0.67 ± 0.07	13.17 ± 3.65
4	7	11.32 ± 2.70	13.63 ± 2.66	0.57 ± 0.08	0.60 ± 0.16	22.72 ± 14.49
5	8	8.48 ± 1.88	9.79 ± 2.05	0.66 ± 0.09	0.83 ± 0.09	5.62 ± 1.75
6	5	7.31 ± 4.36	11.32 ± 6.46	0.74 ± 0.09	0.56 ± 0.08	16.48 ± 6.26
7	9	8.78 ± 3.56	12.58 ± 3.76	0.41 ± 0.06	0.71 ± 0.12	21.17 ± 11.93
8	8	13.51 ± 1.88	17.32 ± 2.14	0.54 ± 0.08	0.71 ± 0.15	22.09 ± 11.27
9	11	12.86 ± 3.58	16.55 ± 7.44	0.45 ± 0.05	0.71 ± 0.09	10.85 ± 2.56
10	10	10.28 ± 4.42	12.16 ± 6.28	0.62 ± 0.10	0.77 ± 0.12	6.27 ± 2.54
Total	67 fruits	10.78 ± 3.77	13.63 ± 5.28	0.53 ± 0.11	0.72 ± 0.13	15.28 ± 10.69
Total	10 shrubs	11.06 ± 2.81	13.85 ± 3.30	0.52 ± 0.08	0.71 ± 0.06	16.00 ± 7.08

*Note*: Means and standard deviation (mean ± SD) are reported within shrubs, and the total between shrubs.

Abreviation: DW, dry weight.

### 3.2 Association between nutrients and defensive metabolites at the intraspecific and intraindividual scales (Objective 2)

**FIGURE 4:**
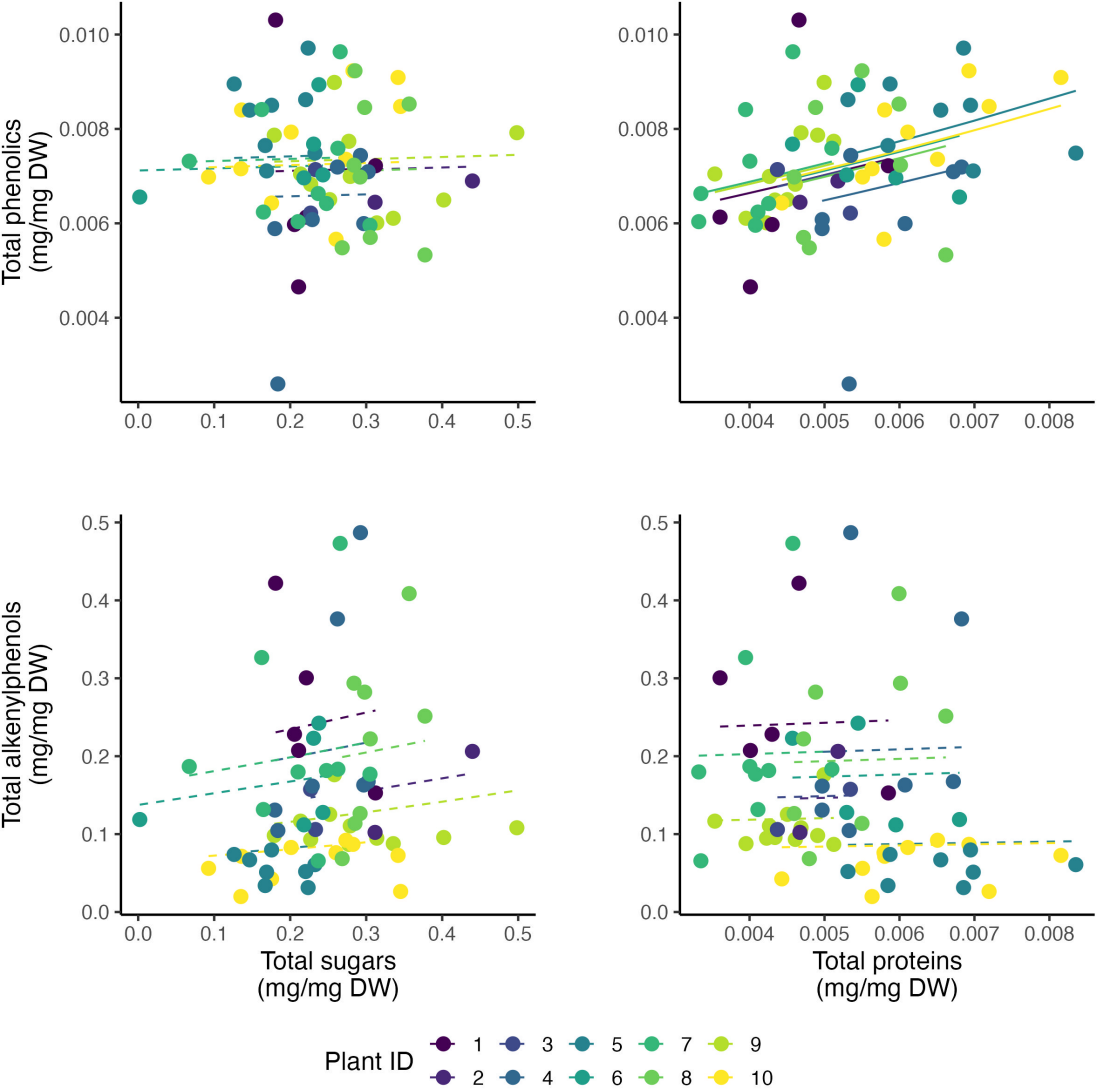
Association between different nutrients and fruit‐defensive metabolites in ripe pulp of *Piper sancti‐felicis* fruits (*N*_fruits_ = 67) at the intraindividual level. Every point represents an individual fruit, colored by plant ID. Black solid lines indicate significant slopes (*p* < .05), whereas dotted lines indicate non‐significant slopes predicted by GLMMs. Parameters estimated by the GLMMs are summarized in Table 2.

**FIGURE 5:**
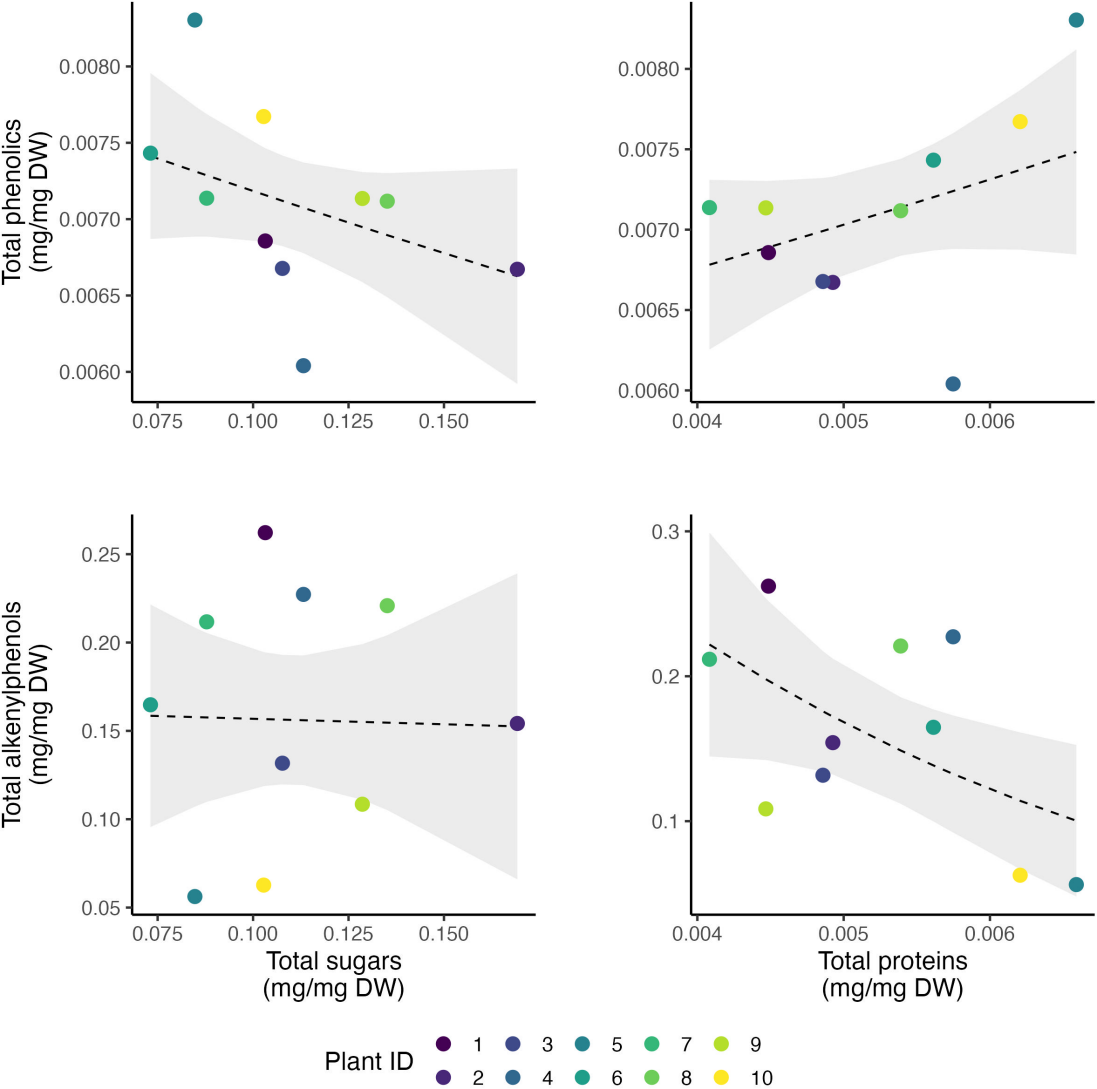
Association between different nutrients and fruit‐defensive metabolites in ripe pulp of *Piper sancti‐felicis* fruits (*N*_*Shrubs*_ = 10) at the intraspecific level. Every point represents the trait average per plant, colored by plant ID. Black solid lines indicate significant slopes (*p* < .05), whereas dotted lines indicate non‐significant slopes predicted by GLMs. Gray areas represent 95% confidence intervals. Parameters estimated by the GLMs are summarized in Table 3.

We found that proteins, but not sugars, were correlated with secondary metabolites, and the relationships varied across scales and the two classes of secondary metabolites examined (Figures 4 and 5). There was a positive significant association between proteins and phenolics at the intraindividual level (Figure 4, Table 2). With a doubling of protein concentration (from 0.004 to 0.008 mg/mg) and keeping total sugars constant, the GLMM predicted a 33% increase in total phenolics (from 0.006 to 0.008 mg/mg). At the intraspecific level (Figure 5, Table 3), the trend was similar, but with weak statistical support; with a doubling of protein concentration (from 0.004 to 0.008 mg/mg) and keeping total sugars constant, the GLM predicted a 16% increase in total phenolics (from 0.005 to 0.006 mg/mg). In contrast, there was a marginally significant negative association between proteins and alkenylphenols, but only at the intraspecific level (Figure 5, Table 3). With a doubling of protein concentration (from 0.004 to 0.008 mg/mg) and keeping total sugars constant, the GLMM predicted a 300% decrease in alkenylphenols (from 0.2 to 0.05 mg/mg). For sugars (glucose and fructose), we did not find any clear pattern of association with either class of secondary metabolites (Figures 4 and 5, Tables 2 and 3).

**TABLE 2:** Output of two GLMMs investigating the association between fruit‐defensive metabolites and nutrients at the intraindividual level.

Response variable	Predictor variable	Coefficient	SE	95% CI (low, high)	*z*	*p*‐Value	Adjusted *p*‐Value
Total phenolics Conditional *R* ^2^ .181 Marginal *R* ^2^ .104	Intercept	−5.240	0.136	−5.506, −4.975	−38.659	<**.01**	
	Total sugars	0.062	0.282	−0.492, 0.615	0.219	.827	1
	Total proteins	55.528	21.976	12.455, 98.600	2.527	**.012**	**.023**
	Intercept, random effect	0.054	NA	0.017, 0.170	NA	NA	
Total alkenylphenols Conditional *R* ^2^ .564 Marginal *R* ^2^ .025	Intercept	−2.105	0.523	−3.129, −1.080	−4.026	<**.01**	
	Total sugars	1.157	0.992	−0.788, 3.101	1.166	.244	.488
	Total proteins	19.027	94.910	−166.992, 205.047	0.200	.841	1
	Intercept, random effect	0.459	NA	0.256, 0.822	NA	NA	

*Note*: In both models, plant ID was included as a random effect. Each defensive metabolite (total phenolics and alkenylphenols) was examined separately. *p*‐values less than .05 are bolded.

**TABLE 3:** Output of two GLMs investigating the association between fruit‐defensive metabolites and nutrients at the intraspecific level.

Response variable	Predictor variable	Coefficient	SE	95% CI (low, high)	*z*	*p*‐Value	Adjusted *p*‐Value
Total phenolics Marginal *R* ^2^ .001	Intercept	−5.018	0.197	−5.405, −4.631	−25.429	<**.01**	
	Total sugars	−1.177	0.846	−2.836, 0.482	−1.390	.165	.330
	Total proteins	39.545	28.734	−16.773, 95.863	1.376	.169	.338
Total alkenylphenols Marginal *R* ^2^ .073	Intercept	0.327	1.193	−2.012, 2.665	0.274	.784	
	Total sugars	−0.473	5.269	−10.800, 9.854	−0.090	.928	1
	Total proteins	−374.515	176.007	−719.484, −29.547	−2.128	**.033**	.066

*Note*: Each defensive metabolite (total phenolics and alkenylphenols) was examined separately. *p*‐values less than .05 are bolded.

## 4 DISCUSSION

In our study, we quantified a suite of chemical traits of ripe fruits that are potentially relevant in the mediation of ecological interactions. We found that for all the chemical traits examined, at least 50% of the variance is explained by intraindividual variation. Also, our results show that proteins, but not sugars, predict secondary metabolite concentration, but these patterns are scale‐dependent. At the intraindividual level, our results show a significant, positive association between total proteins and total phenolics, supporting the “nutrient–toxin titration” and “relative risk hypotheses.” At the intraspecific level, our results show a significant negative association between alkenylphenols and total proteins, supporting the “removal‐rate hypothesis.” Compared to phenolics, alkenylphenols showed a notably high concentration in ripe fruit pulp, which suggests that alkenylphenols play an important role in mediating fruit–frugivore interactions in this species (Maynard et al., 2020).

At least 50% of the variance in the examined chemical traits was explained at the intraindividual level (Figure 3), suggesting that intraindividual chemical variation could have important ecological consequences or adaptive significance. Although some of this variation may be due to measurement error, it was notable that the amount of chemical trait variation differed among individual plants (Figures 2 and 3), suggesting that variability per se could be a target of selection. Recent evidence suggests that intraindividual variability can be a heritable trait, perhaps in large part due to transgenerational epigenetic processes, and may be adaptive in many scenarios (Herrera et al., 2021; Robinson et al., 2022; Sobral et al., 2014). As proposed for morphological variation (Herrera, 2017; Herrera et al., 2015), the intraindividual chemical variation reported here has the potential to increase plant fitness by improving the use of resources in a heterogeneous environment, such as variation in sunlight, water availability, among others. *Piper sancti‐felicis* shrubs grow mainly in open areas and along forest edges, likely generating different microclimates within individual shrubs. In the context of biotic interactions, spatiotemporal variability can increase the effectiveness of chemical defenses by decreasing the potential for herbivores to physiologically acclimate to toxins (Karban, 2020; Pearse et al., 2018). In fruits, this could lead to important trade‐offs, where high intraindividual variability in nutrients and defenses may reduce damage from herbivores, but also deter mutualistic seed dispersers. However, even if variability reduces fruit removal from any single seed disperser, it may still provide benefits in the context of multiple biotic interactions. *Piper* fruits interact with a diverse community of frugivores (Castaño et al., 2018; Fleming, 2004; Maynard et al., 2020), and different sets of chemical traits in fruits may appeal to different frugivores, allowing a bet‐hedging strategy (Childs et al., 2010) for seed dispersal. In a bet‐hedging strategy scenario, heterogeneity in fruit chemical traits might promote fruit consumption by a wider array of frugivores that can disperse seeds from one individual shrub to a larger diversity of sites suitable for germination and decrease competition between frugivore groups. For instance, birds are less deterred by alkenylphenols than bats (Maynard et al., 2020). Birds typically defecate seeds while being perched, while bats defecate during flight, offering different, and perhaps complementary, seed dispersal patterns across a range of microclimates and dispersal distances (Mello et al., 2011; Ripperger et al., 2019).

One key finding from our study is that associations between nutrients and secondary metabolites depend on the class of compounds involved. We found a significant positive association with proteins for phenolics, supporting the “nutrient‐toxin titration/relative risk hypotheses.” Nutritious fruits are likely at high risk of attack from pathogens, which could necessitate a higher concentration of defensive metabolites. At the same time, some seed dispersers might select high‐proteinaceous (Rojas et al., 2021) *Piper* fruit pulp, coping with a relatively higher concentration of total phenolics that serve to defend the fruit against pests, as predicted by the “nutrient‐toxin titration hypothesis.” Although the detoxification capacities of vertebrate frugivores have been little explored, a suite of detoxification enzymes (Foley & Moore, 2005; Lerch‐Henning et al., 2017) and microbes (Kohl et al., 2018) might play a role in coping with high concentrations of fruit secondary metabolites. In contrast, for alkenylphenols, we found a significant negative association with proteins, supporting the “removal‐rate hypothesis.” Once ripe, *P. sancti‐felicis* fruits ripen late in the afternoon and more than 91% are removed that same night by bats (Maynard et al., 2020). If the “removal‐rate hypothesis” explains the negative association between proteins and alkenylphenols in this species, then high‐protein, low‐alkenylphenol fruits should be more likely to be removed as the first option in the early hours of the evening. Models of bat movement suggest that even small delays in fruit removal throughout the evening (<40 min) reduce average dispersal distances by more than 40% because bats forage over longer distances in the early part of the evening (Baldwin et al., 2020). Thus, in *Piper* spp. fruits, variation in shrub strategies that involve removal rate (e.g., high removal/low defense vs. low removal/high defense) may significantly affect seed dispersal outcomes.

The “removal‐rate” and “nutrient‐toxin titration/relative risk hypotheses” assume that secondary metabolites function primarily in fruit defense. However, some compounds may instead be primarily directed to mutualistic seed dispersers (Cipollini & Levey, 1997a). For example, phenolics may function primarily to decrease frugivore protein assimilation in high‐proteinaceous *Piper* fruits, forcing frugivores to find other sources of protein, potentially increasing seed dispersal distances and leading to heterogeneous germination sites (Cipollini & Levey, 1997a). Evidence suggests that phenolics, especially tannins, reduce protein absorption in vertebrates (Constabel et al., 2014; DeGabriel et al., 2009; Mueller‐Harvey, 2006), but the potential for these chemical interactions to drive fruit trait evolution is still unclear. Other secondary metabolites in fruits can affect gut retention time (Baldwin & Whitehead, 2015; Izhaki, 2002; Tewksbury et al., 2008) or deter less effective mutualists (Tewksbury & Nabhan, 2001).

An alternative to the adaptive explanations above is that patterns of covariation between nutrients and secondary metabolites could be driven largely by intrinsic and extrinsic constraints. First, physiological constraints in synthesizing nutrients and defensive metabolites can promote either a positive or a negative association between the two groups of molecules (Pott et al., 2019). Phenolics are synthesized by the phenylpropanoid and the shikimic acid pathway, and the primary precursor is the amino acid phenylalanine (de la Rosa et al., 2019; Saltveit, 2017), establishing a biosynthetic link between phenolics and proteins and potentially promoting the positive association we found. Compared to phenolics, alkenylphenols are less studied, and their biosynthetic pathways have not been elucidated. Second, linkage disequilibrium between the genes that codify for different enzymes involved in synthesizing nutrients and defenses can also promote a positive correlation. Although linkage disequilibrium has been primarily studied in plant morphology (revi ewed in Flint‐Garcia et al., 2003), evidence suggests that linkage disequilibrium also occurs between chemical traits (Bauchet et al., 2017; Otyama et al., 2019). For instance, potential linkage disequilibrium between the enzymes involved in phenolics and protein synthesis may explain the observed positive association reported here. Finally, nutrient availability in the soil can increase the nutrient and defense concentration in plant tissues (Dyer et al., 2004; Glassmire et al., 2022), creating a pattern where plants that grow in sites with more nutrients have higher concentrations of nutrients and defenses. Nutrient availability may also vary within an individual plant based on factors such as branch architecture (Li et al., 2010), but little is known about the extent to which these factors shape within‐plant allocation patterns to nutritional rewards and defense metabolites.

Another key result of our study is that the biological scale at which the association between nutrients and defensive compounds is evaluated can change the strength and direction of the results. In our results, we found a marginally significant negative association between total proteins and alkenylphenols, but this pattern was only at the intraspecific level. One possible explanation for this pattern is that trade‐offs between attraction of seed dispersers and defense against antagonists has led to the evolution of different strategies across individual plants, perhaps driven by different fruit removal probabilities across space. Plants with high potential removal rates may benefit more from a strategy of high nutrient/low defense, whereas plants with lower potential removal rates may benefit from a strategy of low nutrient/high defense. This heterogeneity in removal rates, with certain plants being more visited than others, has been documented in some frugivores (Tonos et al., 2022). Future work examining how patterns of chemical trait variation link to variation in fruit removal and damage rates, within and across shrubs, will further elucidate the mechanisms that explain the varying patterns we observed across scales.

Although we detected some significant associations between proteins and secondary metabolites, we did not observe any clear patterns between sugars and secondary metabolites. This suggests that either the physiological and evolutionary linkages between sugars and secondary metabolites are limited, or the many factors that shape plant chemical traits may obscure potential associations. Different levels of leaf herbivory can modify the allocation of resources to fruit‐defensive metabolites (Whitehead & Poveda, 2011) and nutrient concentrations (Machado et al., 2017). Similarly, the fruit microbial community can potentially degrade or modify nutrients and secondary metabolites (Nevo et al., 2019). Finally, exposure to different abiotic stressors can increase the concentration of soluble sugars and amino acids, as well as the concentration of some chemical defenses (Sardans et al., 2020). Thus, the interaction among plants, herbivores, microbes, and abiotic stressors may lead to non‐proportional changes in nutrients and/or defensive metabolites in plant tissues. As a result, any existing positive or negative association between nutrients and defensive metabolites may be difficult to detect.

Our results show high chemical trait variability and significant associations between nutrients and defensive metabolites in *P. sancti‐felicis* at multiple scales. We illustrate the importance of considering multiscale variability, both within plant populations and within individual plants. The high intraindividual variability found here might play an important role in the ecology of fruit‐frugivore interactions. Furthermore, we found that sugars and defensive metabolites occur mostly independently, whereas proteins are significantly associated with secondary metabolites, finding support for both the “removal‐rate hypothesis” and the “nutrient‐toxin titration/relative risk hypotheses” at different scales. Future studies should evaluate how frugivores respond to the high chemical variation of fruit pulp and to the different associations between chemical traits. Continued exploration of the causes and consequences of variation and covariation in nutritional rewards and defensive metabolites informs us about the ecology and physiology of plants and allows us to better understand how chemical traits mediate ecological interactions among plants, mutualists, and antagonists at multiple scales.

## AUTHOR CONTRIBUTIONS

**Mariana Gelambi:** Conceptualization (equal); data curation (lead); formal analysis (lead); funding acquisition (supporting); investigation (equal); methodology (lead); project administration (lead); writing – original draft (lead); writing – review and editing (equal). **Susan R. Whitehead:** Conceptualization (equal); funding acquisition (lead); formal analysis (supporting); investigation (equal); project administration (supporting); writing – review and editing (equal).

## Data Availability

All data, metadata, and R scripts used to generate results and figures are archived in a Zenodo repository DOI: 10.5281/zenodo.8250520 (Gelambi & Whitehead, 2023).

## APPENDIX A

**TABLE A1:** Retention time (RT), average abundance, and frequency of detection of individual alkenylphenols.

Compound	Letter	RT ± SD	Average abundance (mg/mg DW) ± SD	Frequency of detection
4‐[(3′ E)‐dodec‐3′‐en‐1‐yl]phenol	A	12.518 ± 0.0048	1.68 ± 1.46	45/67 (67.16%)
4‐[(4′ E)‐dodec‐4′‐ en‐1‐yl]phenol	B	12.592 ± 0.0033	3.99 ± 3.31	67/67 (100%)
4‐[(3′,5′,E, E)‐ dodec‐3′,5′‐dienyl‐1‐yl]phenol	C	12.964 ± 0.0026	0.01 ± 0.08	2/67 (2.99%)
4‐[(4′,6′,E, E)‐dodec‐4′,6′‐dienyl‐1‐ yl]phenol	D	13.046 ± 0.0055	0.71 ± 0.72	57/67 (85.07%)
4‐[(3′ E)‐tetradec‐3′‐en‐1‐yl]phenol	E	13.687 ± 0.0039	2.18 ± 1.57	62/67 (92.54%)
4‐[(4′ E)‐Tetradec‐4′‐en‐1‐yl]phenol	F	13.758 ± 0.0048	5.54 ± 3.87	67/67 (100%)
4‐[(4′,6′,E, E)‐dodec‐4′,6′‐dienyl‐1‐ yl]phenol 4‐[(3′,5′,E, E)‐tetradec‐3′,5′‐dienyl‐1‐yl]phenol	G	14.388 ± 0.006	0.53 ± 0.40	52/67 (77.61%)
4‐[(4′,6′,E, E)‐tetradec‐4′,6′‐dienyl1‐yl]phenol	H	14.48 ± 0.003	0.23 ± 0.16	55/67 (82.09%)
4‐[(3′ E)‐hexadec‐3′‐en‐1‐yl]phenol	I	15.179 ± 0.0026	0.28 ± 0.33	45/67 (67.16%)
4‐[(4′ E)‐hexadec‐4′‐en‐1‐yl]phenol	J	15.307 ± 0.0029	0.12 ± 0.12	45/67 (67.16%)
